# Beyond the Fixed Dose in Theranostics: Pragmatic Dosimetry as the Missing Link in Optimizing Precision Nuclear Oncology

**DOI:** 10.1055/s-0046-1827826

**Published:** 2026-08-29

**Authors:** Akram Al-Ibraheem, Kalevi Kairemo

**Affiliations:** 1 Department of Nuclear Medicine, King Hussein Cancer Center (KHCC), Amman, Jordan; 2 Department of Radiology and Nuclear Medicine, School of Medicine, University of Jordan, Amman, Jordan; 3 Department of Theranostics, Docrates Cancer Center, Helsinki, Finland

Radiotheranostics is rapidly evolving from a specialized nuclear medicine application into an important component of precision oncology. The success of [ ^177^ Lu]Lu-DOTATATE and [ ^177^ Lu]Lu-PSMA-617 has demonstrated that molecularly targeted radionuclide therapy can improve clinically meaningful outcomes and has accelerated the development of new therapeutic targets and radionuclides. 1 2 Yet, despite the inherently personalized nature of theranostics, most approved radiopharmaceutical therapies continue to be administered using fixed or weight-based activity schedules derived from population-level clinical trials. This raises an important question: can theranostics fully realize the promise of precision medicine while treatment activity remains largely standardized rather than individualized?

The principle of theranostics is based on personalization. Molecular imaging enables visualization of therapeutic targets and identification of patients most likely to benefit from a given radiopharmaceutical. However, identifying the *right patient* does not necessarily determine the *right amount of treatment* . Patient-specific dosimetry provides the missing quantitative link by estimating the absorbed radiation dose delivered to both tumors and normal tissues. In this context, companion imaging determines *who* should be treated, while dosimetry has the potential to determine *how much* treatment an individual patient should receive. 3

Administered activity and absorbed dose should not be regarded as interchangeable. Patients receiving the same administered activity may experience substantially different tumor and normal-organ absorbed doses because of variations in tumor burden, target expression, organ volume, renal function, biodistribution, and pharmacokinetics. Considerable interpatient variability in renal absorbed dose has been demonstrated among patients receiving standardized [ ^177^ Lu]Lu-DOTATATE treatment. 4 Similarly, lesion-based studies with [ ^177^ Lu]Lu-PSMA have demonstrated substantial variability in tumor absorbed dose and clinically meaningful relationships between absorbed dose and treatment response. 5 These observations underscore a fundamental limitation of fixed-activity treatment: standardizing the administered activity does not standardize radiation exposure.

This contrasts with external beam radiotherapy and brachytherapy, in which absorbed-dose calculation is integral to treatment planning. Radiopharmaceutical therapy is unquestionably more complex because radiation delivery depends on biological distribution and clearance over time. Nevertheless, absorbed dose remains the physical quantity most directly related to radiation energy deposition, and accumulating evidence indicates that it can serve as a relevant biomarker of therapeutic effect in radiopharmaceutical therapy. 3 In [ ^177^ Lu]Lu-PSMA therapy, tumor absorbed dose has been associated with biochemical response and other clinically relevant outcomes, further supporting the potential value of dosimetry in therapeutic decision-making. 5

Importantly, the purpose of dosimetry should not simply be to calculate and report absorbed dose after treatment. Its real clinical value lies in making that information actionable. Dosimetry may identify patients approaching normal-organ tolerance, but equally importantly, it may identify patients receiving relatively low normal-tissue doses who could potentially tolerate treatment intensification. Personalized dosimetry should therefore not be regarded merely as a radiation-protection exercise. Its ultimate objective is optimization of the therapeutic index: delivering sufficient radiation to tumors while maintaining acceptable exposure to organs at risk.

This concept is increasingly reflected in contemporary practice recommendations. The joint EANM/SNMMI guideline for [ ^177^ Lu]Lu-PSMA radioligand therapy recognizes the importance of quantitative assessment and dosimetry within the broader framework of safe and individualized radiopharmaceutical therapy. 6 At the same time, prospective and retrospective experience in peptide receptor radionuclide therapy has demonstrated that patient-specific dosimetry can permit individualized treatment while maintaining acceptable normal-organ exposure. 3 4

One of the major barriers to widespread implementation has been the perception that dosimetry is too complex and resource-intensive for routine clinical practice. Conventional protocols may require several quantitative SPECT/CT acquisitions over multiple days, followed by image registration, segmentation, time–activity curve fitting, and absorbed-dose calculations. 3 These requirements impose additional burdens on patients, scanners, technologists, physicians, and medical physicists. However, this landscape is changing rapidly. Simplified single- and dual-time-point protocols, improved quantitative SPECT/CT, automated segmentation and registration, and increasingly sophisticated dosimetry software are substantially reducing the workload associated with patient-specific calculations. 7

The field should therefore avoid allowing the pursuit of dosimetric perfection to become a barrier to clinically useful dosimetry. Comprehensive multi-time-point and voxel-level approaches remain important, particularly for research, radiopharmaceutical development, unusual pharmacokinetics, and complex clinical situations. However, appropriately validated simplified protocols may provide sufficient information for routine clinical decision-making. Rapid predictive approaches, including “first-strike” dosimetry, further illustrate the possibility of moving dosimetric information earlier in the therapeutic pathway and potentially informing activity prescription before or during treatment. 8 This pragmatic approach is particularly relevant in pediatric nuclear medicine, where individualized dosimetry must balance accurate activity prescription and organ protection against the additional burden of prolonged multi-time-point imaging, including motion, sedation, and logistical considerations. 9 The objective should be a pragmatic balance between accuracy, clinical relevance, patient burden, and available resources.

At the same time, dosimetry itself must evolve alongside radiotheranostics. Organ-level mean absorbed dose cannot fully describe the spatial heterogeneity of radiation deposition within tumors and normal tissues. Lesion- and voxel-level approaches increasingly provide three-dimensional information about absorbed-dose distribution and may improve our understanding of heterogeneous treatment response. Integration with quantitative imaging, pharmacokinetic modeling, radiomics, and artificial intelligence could ultimately transform dosimetry from retrospective dose calculation toward prospective treatment-response prediction and adaptive activity prescription. 10

The emergence of targeted alpha therapy introduces additional challenges. Dosimetry for ^225^ Ac and other alpha emitters cannot simply reproduce the framework developed for ^177^ Lu. Their short particle range, high linear energy transfer, complex decay chains, daughter redistribution, low administered activities, and limited quantitative imaging statistics make conventional macroscopic absorbed-dose estimation considerably more difficult. 11 Consequently, microdosimetry, spatial energy deposition, relative biological effectiveness, and decay-chain-specific modeling will become increasingly relevant as targeted alpha therapy matures. The future question may therefore extend beyond *how many Gy were delivered?* to asking *where the energy was deposited, and with what biological effectiveness* ?

Implementation must also take global realities into account. Theranostic capacity remains unevenly distributed worldwide, with substantial differences in imaging infrastructure, radionuclide availability, medical physics support, workforce, regulatory pathways, and reimbursement. 12 13 Sustainable expansion of radiotheranostics, particularly in low- and middle-income countries, requires not only access to radiopharmaceuticals but also appropriate training, quality management, infrastructure, and scalable dosimetry frameworks. 12 13 Requiring highly resource-intensive dosimetry for every treatment could unintentionally widen existing inequalities. Conversely, abandoning dosimetry because the most sophisticated methodology is not universally feasible would represent another missed opportunity.

A more appropriate strategy is therefore to adopt scalable dosimetry. Comprehensive approaches should remain available where clinically necessary, while simplified and validated workflows should facilitate broader routine implementation. Professional societies and international organizations have an important role to play through harmonized protocols, workforce training, quality assurance, multicenter validation, and practical implementation guidance. This approach would allow dosimetry to expand in parallel with theranostics rather than becoming an additional barrier to access.

Fixed-activity regimens will remain important because they are supported by randomized clinical evidence, facilitate reproducibility, and enable widespread treatment delivery. 1 2 Dosimetry should therefore complement rather than challenge these achievements. However, population-derived administered activity should probably be regarded as the starting point rather than the final destination of precision radiopharmaceutical therapy.

The next phase of theranostics should move beyond asking whether the appropriate molecular target is present. We should increasingly ask whether sufficient radiation has reached the tumor, whether normal tissues can safely tolerate additional treatment, and whether subsequent cycles should be adapted according to the individual patient's dosimetric and biological response. Achieving this transition will require stronger prospective dose–response evidence, standardized methodologies, simplified workflows, automation, appropriate reimbursement, and multidisciplinary collaboration.

Theranostics has already brought us closer to delivering the right radiopharmaceutical to the right patient. Dosimetry offers the opportunity to complete this paradigm by delivering the right absorbed dose. This should be the next step toward truly personalized radiopharmaceutical therapy.
